# Emotion Recognition in Cats

**DOI:** 10.3390/ani10071107

**Published:** 2020-06-28

**Authors:** Angelo Quaranta, Serenella d’Ingeo, Rosaria Amoruso, Marcello Siniscalchi

**Affiliations:** Department of Veterinary Medicine, Animal Physiology and Behavior Unit, University of Bari “Aldo Moro”, 70121 Bari, Italy; serenella.dingeo@uniba.it (S.d.); rosy.amoruso@gmail.com (R.A.); marcello.siniscalchi@uniba.it (M.S.)

**Keywords:** cat, emotion, behavior, physiology, animal welfare

## Abstract

**Simple Summary:**

The ability to perceive other individuals’ emotions plays a central role for animals living in social groups. Cats entertain social relationships with individuals of the same species (conspecifics) as well as with humans (heterospecifics). Although previous studies have demonstrated that cats are sensitive to conspecific and human communicative signals, their perception of these species’ emotions hasn’t been extensively investigated. In the light of this, the aim of the present work was to investigate cats’ ability to recognize conspecific and human emotions. Our results demonstrate that cats integrate visual and auditory signals to recognize human and conspecific emotions and they appear to modulate their behavior according to the valence of the emotion perceived. The understanding of cats’ socio-cognitive abilities to perceive their close partners’ emotions is crucial for improving the quality of human-cat and cat-cat relationships as well as cat welfare in the domestic environment.

**Abstract:**

Recent studies demonstrated that cats form social bonds with both conspecifics and humans. One of the key factors regulating social interactions is the transfer of emotions between the individuals. The present study aimed at investigating cats’ spontaneous ability to match acoustic and visual signals for the recognition of both conspecific and human emotions. Different conspecific (cat “purr” and “hiss”) and heterospecific (human “happiness” and “anger”) emotional stimuli were presented to the tested population using a cross-modal paradigm. Results showed that cats are able to cross-modally match pictures of emotional faces with their related vocalizations, particularly for emotions of high intensity. Overall, our findings demonstrate that cats have a general mental representation of the emotions of their social partners, both conspecifics and humans.

## 1. Introduction

The recognition of individuals is central in social species. Faces and voices convey information about individual identity and represent the most relevant cues used by human and several non-human species for individual recognition [1,2,3,4,5]. Recent studies have reported that some animals have an efficient visual (cattle: [6]; sheep: [7]; horses: [8]; and dogs: [9]) and auditory recognition of their conspecifics (cats: [10]; dogs: [11,12]; cattle: [13]; pig: [14]; and horses: [15]). Several species of domestic mammals are also able to discriminate between familiar and unfamiliar humans (cats: [16]; pigs: [17]; cattle: [18]; and horses: [4]) and form a memory of specific persons that influence their reactions in subsequent interactions (pigs: [19]; and horses: [20,21]). Moreover, a growing body of literature has demonstrated that domestic species recognize human faces (dogs: [5]; sheep: [22,23]; horses: [24]) and voices (cats: [3]; horses: [20,21]; pig: [19,25]; and dogs: [26]). Animals not only identify conspecifics and humans through separate sensory modalities (e.g., cats: [3]; dogs: [5]; goats: [27]; sheep: [22,23]; cattle: [6]; and cheetahs: [28]) but they are also capable of integrating identity cues from multiple sensory modalities to recognize them (dogs: [26,29]; horses: [30,31]; goats: [32]; rhesus monkeys: [2,33]; crows: [34], and cats: [35]). This high-level cognitive ability demonstrates that animals form a multimodal internal representation of individuals that is independent of the sensory modality [36]. It allows an accurate and reliable recognition of other individuals, since inputs from a single sensory domain are combined with the stored information previously acquired from other sensory modalities in order to activate a cognitive representation in the animal’s favored modality [37,38]. Cross-modal recognition has been recently found in goats [32], horses [39], dogs [40], crows [34], and non-human primates [33,41] for conspecific identification. Interestingly, species living in close contact with humans are also capable of integrating visual and auditory cues to identify a familiar human, as recently demonstrated in horses [30,31], rhesus monkeys [2], and dogs [26], which also generalize this ability to unfamiliar people [29]. A recent study has provided evidence about cats’ cross-modal ability to recognize humans by matching individual voices and faces. However, contrary to the above-mentioned species, this ability appears to be limited to the owners and it is not extended to unfamiliar people. Cats, indeed, can predict the owners’ face upon hearing their voices. In addition, cats’ lifetime experience with humans seems to affect their ability to cross-modally recognize them [35]. Therefore, further studies are needed to shed light on cats’ ability to integrate sensory information to recognize conspecifics and unknown humans. Cats, along with dogs, are the most popular pets, yet their socio-cognitive abilities remain poorly investigated and understood. The scientific interest shown towards dogs in the last decades, indeed, has been considerably higher compared to cats [42]. Having shared the same living environment with humans for at least 10,000 years [43], cats entertain complex and long-lasting relationships with their owners [44,45] that have been recently classified as attachment bonds [46], as previously described for the human-dog relationship [47,48]. During domestication, they became sensitive to human communicative signals and developed human-compatible social skills that enable them to communicate with humans. Cats successfully use human pointing gestures to locate hidden food [45] and follow the human gaze for referential information [49]. Moreover, they exhibit referential looking toward their owner in order to receive information about a novel and potentially frightening object [50]. They also discriminate between familiar and unfamiliar humans [16] and distinguish the voices of their owners from strangers [3]. A recent study has also shown that cats discriminate their own names from general words when pronounced both by their owner and unfamiliar persons [51]. However, to date, cat emotional communication with both conspecifics and humans has received limited attention.

In social species, emotional recognition plays a pivotal role, since emotions regulate social interactions. In particular, the transfer of emotion in domestic animals is not only related to conspecifics, but also occurs during human-animal interactions. It has been found, for instance, that dogs and horses perceive the content of human and conspecific emotional signals through single sensory modalities (i.e., they perceive human and conspecific emotional vocalizations [11,52,53], faces [54,55], and olfactory signals [56], see [57] for review), but also cross-modally [30,39,40], suggesting that they form a cognitive multimodal representation of other individuals’ inner states. Moreover, dogs and horses show a functional understanding of human emotional signals and adjust their behavior according to the valence and intensity of the emotional message conveyed [11,52,53,54,55,56,57,58,59]. Similar results have been recently reported by Nawroth and colleagues for goats, which discriminate human facial expression of anger and happiness and prefer to interact with the latter [60]. Studies on cats showed that they are sensitive to conspecific and human emotional signals, though to a lesser extent than dogs [10,42,50]. They discriminate between human emotional cues, which, however, produce only slight and subtle changes of cat behavior in accordance with the owner’s emotional expressions [42,50]. Moreover, it has been found that cats are sensitive to human moods, and in particular, they engage more frequently in social interactions with depressed humans [61] and approach more frequently owners feeling extroverted or agitated [62].

In light of this evidence, the present study aims at investigating if cats are able to integrate visual and auditory signals to recognize human and conspecific emotions and if they modulate their behavior according to the valence of the emotion perceived. In order to test these hypotheses, we studied cat cross-modal recognition of the emotional signals of conspecific “hiss” and “purr” vocalizations and human “happiness” and “anger” expressed by facial expressions and their related vocalizations. We predicted that if cats can cross-modally recognize conspecific and human emotions, they would look longer at facial expressions matching the vocalization just heard. Moreover, if they possess a functional understanding of conspecific and human emotions, they would show different level of stress according to the valence of the emotion perceived, namely, a higher stress level in response to cat “hiss” and human “anger” than for the other stimuli.

## 2. Materials and Methods

### 2.1. Participants

The study population was composed of ten domestic cats, 6 males and 4 females, all neutered, whose ages ranged between 2 to 10 years (5.3 ± 2.41; mean ± s.d.). All subjects were pets living in 10 separate households and were experimentally naïve. Cats belonged to feline colonies living in the urban environment going back generations. They were adopted within 6 months of age and lived in a human social group (made up of both men and women) from at least 3 years of age (see Table 1). Moreover, each subject lived in a house with a garden and had daily interaction with the conspecifics living nearby. The experiment was carried out in their living environment, namely at their house, in order to avoid any potential influences of location novelty on the cats’ stress and vigilance levels.

### 2.2. Emotional Stimuli

**Figure 1:**
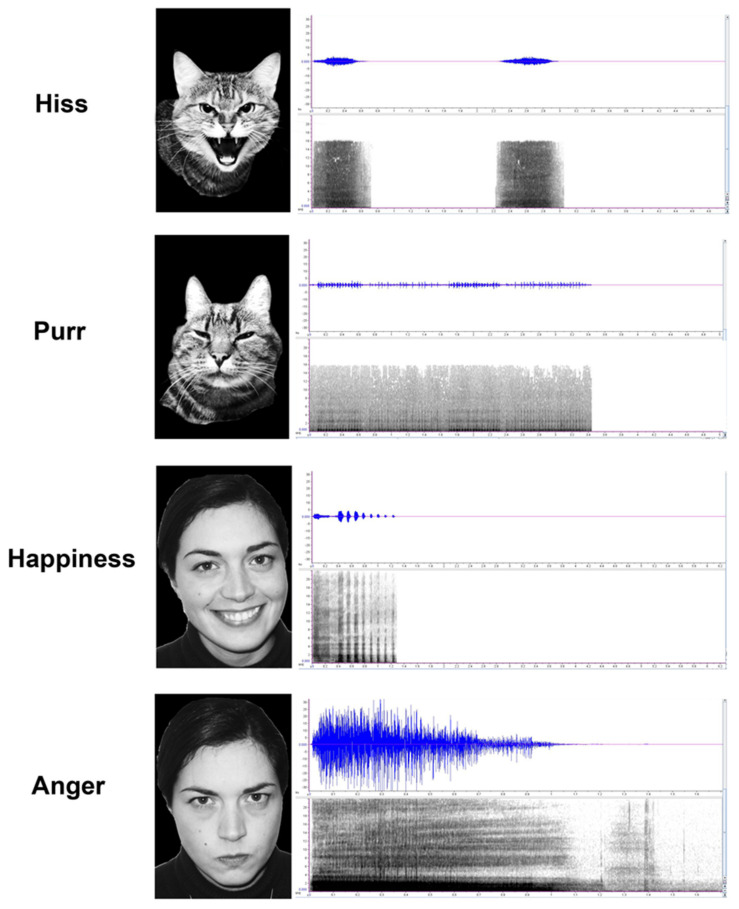
Examples of faces and their corresponding vocalizations used in the cross-modal paradigm (“human-anger” vs “happiness”; “cat-hiss” vs “purr”). Sonograms show the different emotional vocalizations (time in seconds, frequency measured in kHz).

Two human volunteers, a man and a woman, aged 29 and 24 years old, respectively, were photographed while posing facial expressions of “happiness” and “anger” [54]. They had to remove any make-up and facial hair, as well as glasses, piercings, and earrings that could be used by cats as cues to distinguish between emotional facial expressions. The human volunteers were also asked to pronounce non-verbal vocalizations of “happiness” (laughs) and “anger” (growls) [52,53,63], following the procedure previously described by Siniscalchi et al. [52] (see Figure 1).

For the conspecific stimuli, vocalizations and facial expressions of two cats, a male and a female (both neutered), aged 16 and 11 years old, respectively, were collected during two different emotional events: a stressful situation, caused by a dog approaching the place where the cats were resting, and a relaxed situation, in which cats were petted by their owners. The acoustic and visual signals of “hiss” and “purr” were therefore obtained (see Figure 1). Hence, a total of eight emotional stimuli (4 × human and 4 × conspecific stimuli) were finally obtained. Moreover, a neutral sound for the control condition (“Brownian sound”) was obtained from the web.

The conspecifics and human vocalizations were digitally recorded using Roland Edirol R-09HR, at a 24-bit quantization and 96 kHz sampling rate, in mono. They were then edited employing Audition 2.0 (Adobe Inc., San Jose, USA, California) to remove background noises and to homogenize their loudness to 69 dB when measured from the cats’ position in the testing area [52]. As for the visual stimuli, the conspecific and human facial expressions were captured employing a full HD digital camera (Sony Alpha 7 II ILCE-7M2K^®^, SONY, Tokyo, Japan) positioned about 2 m from the subjects. All the pictures were then edited using Adobe Photoshop (Adobe Inc., San Jose, USA, California) in order to homogenize their sizes and to add a uniform black background [54]. Moreover, the pictures were converted to a grayscale to avoid any influence of color and brightness in the choice task [40].

### 2.3. Experimental Setup

**Figure 2:**
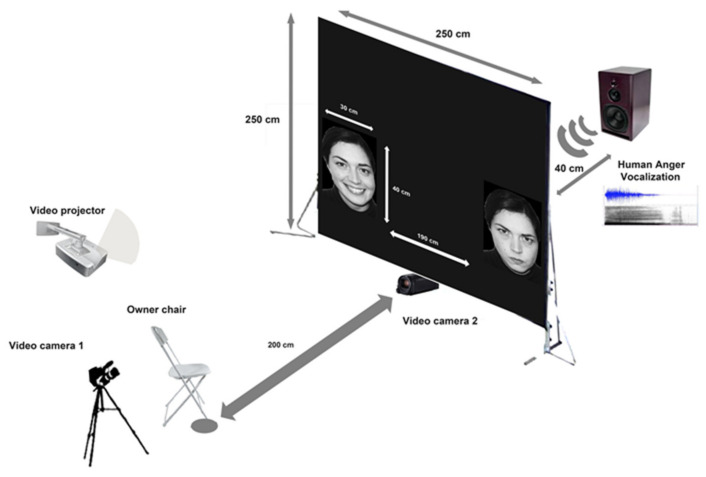
Schematic representation of the testing apparatus.

The experiment was carried out in an isolated room of the house to avoid any noise interference. Visual stimuli (30 × 40 cm) were projected onto a white screen (size 2.5 × 2.5 m) by a WiMiUs TK1000^®^ (WiMiUS, Shenzhen, China) projector placed at a distance of 2 m from the screen (Figure 2). A loudspeaker (FBT-200W8RA^®^, FBT Elettronica SpA, Recanati (MC), Italy) connected to a sound mixer was used to broadcast the emotional vocalizations and it was located centrally and behind the screen. A chair for the cats’ owner was positioned centrally and in line with the speaker, at a distance of 2 m.

Two digital video-cameras were used to record the cats’ behavioral responses when presented with the emotional stimuli. They were positioned on a tripod behind the owner and under the screen, centrally and facing the subjects, in order to register subjects’ spontaneous behavior.

### 2.4. Procedure

Each subject was presented with the emotional stimuli while sitting on their owners’ legs. The test consisted of 3 trials (one per day) with a 2-day inter-session interval. In each trial, two presentations (one per each species) were presented to each subject. Stimuli were presented using the preferential looking paradigm. Therefore, each emotional vocalization (or neutral sound) was simultaneously presented with two different emotional facial expressions of the same species at the two sides of the screen, one matching the emotion expressed by the acoustic stimulus (congruent) and the other displaying the same individual but with a different emotional expression (incongruent). After a pilot test, we decided to abandon multiple presentations of the same stimulus since habituation to the experimental procedure occurred very quickly. Moreover, for the control trials, we presented a neutral sound (“Brownian sound”) paired with the two emotional facial expressions of both cats and humans. Before the test, in the absence of cats, the experimental set-up and the different visual and acoustic stimuli were shown to the owners to avoid unexpected reactions during the trials. Furthermore, during the test, owners were asked to not interact with their cats and to look straight at the screen.

Emotional stimuli were presented as a PowerPoint slideshow in which the first, the last, and in between stimuli slides were a homogeneous black. Each stimuli presentation was preceded by a “beep” sound in order to turn the cats’ attention to the screen. Once the cats were gazing at the screen, the stimuli were presented and remained for 5 s. The inter-stimulus interval was 2 seconds. Visual stimuli were displayed at the bottom of the screen and at its two opposite sides (Figure 2). Two experimenters controlled the stimuli presentation from an adjacent room using a close-circuit video system previously described by Siniscalchi et al. [52].

### 2.5. Ethical Statement

The experiments were conducted in accordance with directive 2010/63/EU of the European Parliament and of the European Council and were approved by the Department of Veterinary Medicine’s (University of Bari) Ethics Committee, EC (Approval Number: 19/18). In addition, before the experiment began, informed consent was obtained from all the participants included in the study.

### 2.6. Data Analysis

#### 2.6.1. Looking Preference

The cats preference to look at different emotional stimuli were computed using the index: CI = (C − I/C + I), where C and I indicate the total time (in s) spent looking at the congruent (facial expression matching emotional vocalization) and incongruent faces during the experiment, respectively. Hence, a score of 1.0 indicated an exclusive look at the congruent face and a score of −1.0 indicated an exclusive look at the incongruent face. A score of 0 indicated equal congruent and incongruent looking preference. A binomial GLMM analysis (General Linear Mixed Model) was performed to assess the influence of “emotion category”, “vocalization gender”, “sex”, and “age” on the test variable “looking preference”, with “subjects” as a random variable. Fisher’s least significant difference (LSD) pairwise comparisons were performed to detect differences between the emotion categories. In addition, asymmetries at group-level (i.e., emotion category) were assessed via a two-tailed one-sample *t*-test, to report significant deviations from zero (i.e., significant departures from chance level). The normality assumption of data was verified both graphically and using the Shapiro-Wilk test.

After a pilot test, we decided to avoid multiple presentation of the same acoustic stimulus, since habituation to emotional vocalizations occurred very quickly.

#### 2.6.2. Behavioral Score

The cats’ behavior was video recorded continuously throughout the experiment. A total of 20 behaviors related to stress/anxiety were considered (see Supplementary Table S1 for the list of the behavior considered). Scores for stress/anxiety behaviors were computed allocating a score of 1 for each behavior displayed (i.e., behavioral scores). For both looking preference and behavior scores, video footage was analyzed by two trained observers, who were blind to the testing paradigm and the stimuli presented. The inter-observer reliability was assessed by means of independent parallel coding of videotaped sessions and calculated as percentage agreement, which was always higher than 94%. GLMM analysis was performed to assess the influence of “emotion category”, “vocalization gender”, “sex”, and “age” on the test variable “stress-behaviors”. To detect differences between the emotion categories, Fisher’s least significant difference (LSD) pairwise comparisons were performed.

Statistical analyses were performed using SPSS software version 22 (IBM, Armonk, USA, New York). Results were considered significant at *p* < 0.05.

## 3. Results

### 3.1. Looking Preference

**Figure 3:**
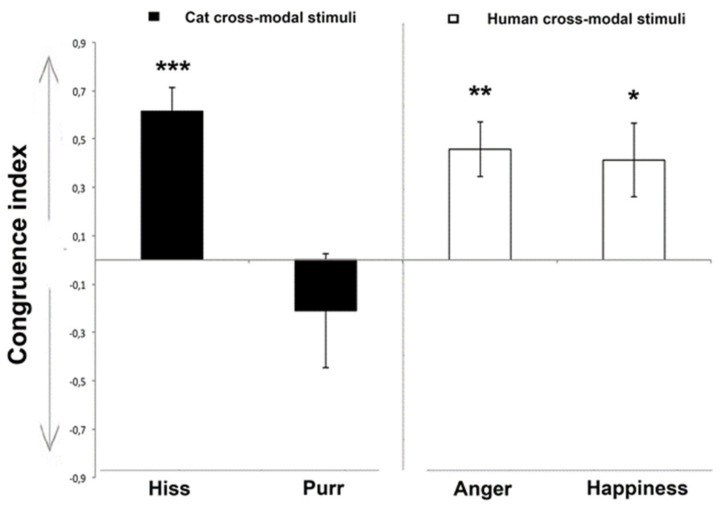
Congruence index for the cross-modal matching of each cat during the experiment: a score of 1.0 represents an exclusive look at the congruent emotional face and −1.0 represents an exclusive look at the incongruent face (group means with SEM are shown). Asterisks indicate significant biases. * *p* < 0.05; ** *p* < 0.01; *** *p* < 0.001 (One-Sample t test).

Results for the cats’ looking preference at visual stimuli are shown in Figure 3. A significant main effect of different facial expression was observed (F(3,33) = 4.212, *p* = 0.013, GLMM analysis). Pairwise comparisons revealed that this main effect was due to “cat-purr” stimuli being significantly different from “cat-hiss”, “human-anger” (*p* < 0.01), and “human-happiness” (*p* < 0.05).

In addition, separate analysis for different emotional faces revealed that for “cat-hiss” (t(9) = 6.275, *p* = 0.000), “human-anger” (t(9) = 3.972, *p* = 0.003), and “human-happiness” (t(9) = 2.693, *p* = 0.025) the mean congruence index was significantly greater than zero (one-sample *t*-test), indicating that cats looked significantly longer at the face whose expression matched the valence of vocalization.

Finally, there was a statistically significant effect of age on the looking preference response (F(1,33) = 6.923, *p* = 0.013, GLMM analysis), showing that younger cats (2–3 years old) (0.60 ± 0.141; mean ± s.d.) have higher mean congruence index values than adult subjects (5–9 years old) (0.178 ± 0.084; mean ± s.d.). No other statistically significant effects were observed regarding looking preference response: “cat gender” (F(1,33) = 0.831, *p* = 0.369); or “vocalization sex” (F(1,33) = 1.140, *p* = 0.293, GLMM analysis).

The cats did not preferentially look at any of the facial expressions shown in the control conditions in which the “Brownian sound” was broadcast (mean preferential looking index for human facial expressions: 0.32 ± 0.15; Z = 14.00, *p* = 0.074; mean preferential looking index for cats facial expression: −0.16 ± 0.14; Z = 1.00, *p* = 0.276, Wilcoxon signed rank test).

### 3.2. Behavioral Score

**Figure 4:**
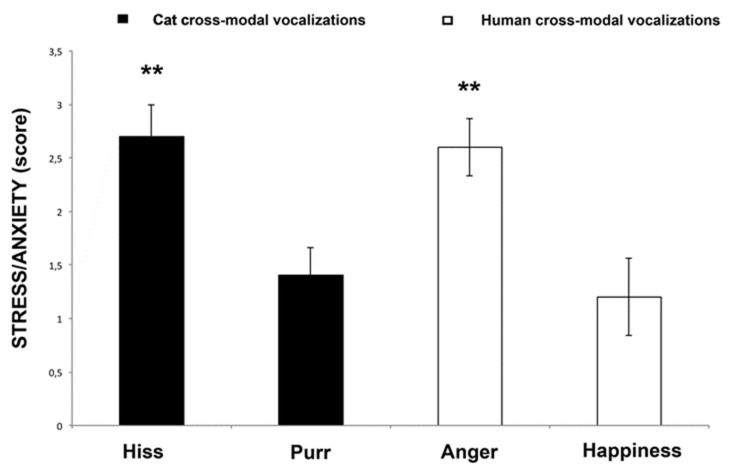
Behavioral score. Data for the score of the stress behaviors for each cat during cross-modal presentation of different stimuli (means with SEM are shown). Asterisks indicate statistical significance according to Fisher’s LSD test. ** *p* < 0.01.

Concerning the behavioral scores, the analysis revealed a significant difference between the emotion category (F(3,33) = 6.491, *p* = 0.001, GLMM analysis, see Figure 4), after controlling for the effect of vocalization gender (F(1,33) = 0.079, *p* = 0.781), sex (F(1,33) = 0.128, *p* = 0.723) and age (F(1,33) = 0.645, *p* = 0.428). Post-hoc pairwise comparisons showed that the cats displayed more stress-related behavior when they responded to “human-anger” and “cat-hiss” emotional stimuli than to all the other stimuli (“human-anger” vs. “human-happiness” (*p* = 0.005) and “cat-purr” (*p* = 0.003); “cat-hiss” vs. “human-happiness” (*p* = 0.005) and “cat-purr” (*p* = 0.003). No statistically significant differences were found between “human-happiness” and “cat-purr” (*p* = 0.683), and between “cat-Hiss” and “human-anger” (*p* = 0.801) (Fisher’s LSD).

## 4. Discussion

We have provided evidence about cats’ ability to recognize cross-modally conspecific and human emotional expressions. Cats spontaneously looked at the congruent facial expressions for longer when hearing the conspecific emotional vocalizations of “hiss” and human emotional vocalizations of “happiness” and “anger”, suggesting that they integrated visual and auditory signals into a cognitive representation of conspecifics’ and humans’ inner states. Moreover, the behavioral results demonstrated that cats respond in a functional way to human “anger” and conspecific “hiss” emotions, since behavioral expression of their stress levels were higher when responding to these emotional stimuli than in response to human “happiness” and conspecific “purr”.

These findings suggest that cats recognize and interpret the emotional signals of the members of their social groups, both conspecifics and humans. Cats, indeed, engage in social behavior and form long-lasting bonds with humans [46,64], which are modulated by individuals’ emotions. Therefore, it is possible that during domestication, cats developed socio-cognitive abilities for understanding human emotions in order to respond appropriately to their communicative signals. This hypothesis is supported by recent finding demonstrating similar skills in other two domestic species living in close contact with humans, namely dogs [52,54,56] and horses [53,55]. It also suggests that the ability to perceive others’ emotions has an adaptive and central role for human-animal interactions and relationship.

The cats’ reactions to the conspecific “hiss” were expected, since it has previously been found that cat agonistic vocalizations (i.e., growls) elicited an increase in receivers’ stress levels [10]. In intraspecific communication, both growls and hisses are used “to signal danger or to warn or scare off an opponent” and often merge together during agonistic interactions [65]. Thus, the high stress levels registered in response to conspecific “hiss” vocalizations and facial expressions suggest that cats perceived these emotional signals as alarming and potentially threatening.

It is interesting to note that cats showed no clear cross-modal recognition of the conspecific “purr” emotion. The lack of significant cross-modal matching of visual and auditory signals of purr could be explained by the several and different biological functions of purring in cats. Purring, indeed, can be observed in social contexts, during interactions with humans, conspecifics, or kittens [64,66], but also in non-social contexts, as anecdotally reported by cats’ owners. Moreover, purrs differ in their functional meaning, since they can communicate cats’ contentment, hunger, stress, and pain, according to the context of their production [65]. In addition, it has been shown that cats alter the acoustical features of their purr to change the meaning of this vocalization [67]. Therefore, the high variability of the meaning and the context of production could explain the cats’ difficulty to recognize and match the facial expressions and vocalizations used in our study. An alternative and complementary explanation for the weak preferential looking bias toward the congruent facial expressions here observed could be found in the higher salience that cats attributed to the conspecific “hiss” facial expression that was simultaneously presented on the screen. The latter could have elicited a higher level of vigilance in the test subjects, diverting their attention from the congruent picture. Another possibility is that the cats perceived the communicative meaning of the purr vocalizations presented in our study, which were produced during cat-owner interactions and, therefore, used for communicating with humans. This may have produced a lower interest toward the congruent conspecific face than toward the “hiss” facial expression. Nevertheless, further studies are needed to investigate the different acoustic features of purrs and the differences in cats’ corresponding facial expressions according to the context of their production.

Regarding the heterospecific emotional signals, we found that cats correctly matched the human auditory and visual signals of “happiness” and “anger”, suggesting that they have a cognitive representation of these emotions, which allow cats to discriminate between them. This is in line with recent findings about cats’ ability to cross-modally recognize humans [35]. Moreover, our results are consistent with previous studies demonstrating that cats are sensitive to human communicative cues [10,50] and to their emotions, particularly if expressed by their owners [16,42]. Cats, indeed, discriminate their owner’s emotional reaction toward an unfamiliar object and adjust their behavior accordingly, expressing more positive behaviors and spending a longer time in contact with their owner when they appeared happy, whereas they displayed less positive behaviors in response to the owner’s angry expression [42]. Moreover, cats moved more quickly and looked for a possible exit when the owner reacted in a fearful way to an ambiguous object [50]. Researchers have suggested that, although cats are sensitive to their owner’s emotional reactions, they only display subtle behavioral differences according to the message conveyed. Our results show, instead, a significant difference in subjects’ stress levels when attending to human “happiness” and human “anger” emotional signals, which were higher in response to human “anger” voices and faces. These findings suggest that cats perceived the negative valence of the human “anger” emotion and responded in a functionally relevant way. Similarly, domestic dogs (*C. familiaris*) and domestic horses (*E. caballus*) showed a functional understanding of human anger emotional signals, which produced an increase in subjects’ arousal and stress/vigilance levels [52,53,54,55]. This suggests the existence of shared mechanisms and a common ability of domestic animals to respond appropriately to human negative emotional stimuli that could have a high adaptive value, since it allows individuals to anticipate and avoid potential negative consequences [55].

Regarding “happiness” emotional signals, we found that cats displayed less stress behaviors than to anger emotional signals. This finding is consistent with those reported for dogs and horses, which showed a positive perception of human vocalizations of happiness and low stress levels in the receivers [52,53]. However, although the low stress levels here found may suggest that cats perceived human happiness signals as non-threatening and potentially positive, further studies are needed to investigate the valence that cats attribute to human “happiness” emotions.

The lack of a significant bias in the cats’ looking preference in response to the acoustic stimulus “Brownian sound” further confirmed that cats have a cognitive representation of emotions of both conspecifics and humans, which allows them to correctly match visual and auditory signals for emotional recognition.

Our results, together with those of previous studies on dogs and horses [52,53,54,55,56,58], indicate that domestic animals’ ability to perceive human emotions could be a phylogenetic product of sharing the same living environment with humans.

Contrary to previous studies showing that cat sensitivity to human emotional cues is restricted to the owner’s (familiar) emotional expressions [41,49], as well as their cross-modal ability to recognize humans [35], we found that cats are able to recognize and interpret unfamiliar human emotional signals, suggesting that they have a general mental representation of humans and their emotions. This cognitive representation, therefore, is pre-existing and is not affected by individual lifetime experiences with humans, as further suggested by the higher ability of younger cats (2–3 years old) to cross-modally recognize human emotions. We therefore hypothesized that cross-modal recognition of individuals could be innate in domestic cats. In other words, this ability could depend on individuals’ phylogeny rather than their ontogeny. In the future, it would be interesting to test a wider population of subjects with a wider range of stimuli to verify the effects of breeds and different living environments (e.g., the possibility of interacting with more cats, the number of interactions with humans) on the cats’ ability to recognize and perceive both conspecific and human emotions.

## 5. Conclusions

Overall, our results showed that cats are able to integrate acoustic and visual emotional signals of a conspecific “hiss” and human “anger” and “happiness”. They also show a functional understanding of highly arousing emotions (i.e., cat “hiss” and human “anger”), regardless of the species that produced them. These findings demonstrate that cats have developed social skills that allow them to understand human emotional signals, which is a key factor for the maintenance of interspecies relationships and for strengthening the human-cat bond.

## Figures and Tables

**Table 1:** Age of adoption and current age of the tested cats

Subjects	Age of Adoption	Current Age (Years)
1	5 months	11
2	1 week	4
3	1 month	3
4	1 month	3
5	4 months	8
6	1 week	7
7	1 week	6
8	1 week	6
9	6 months	7
10	2 months	9

## Supplementary Materials

The following are available online at https://www.mdpi.com/2076-2615/10/7/1107/s1, Table S1: List of behaviors scored according to the Stress/Anxiety category.
