# Supplementary material for: Emotion Recognition in Cats
Source: Animals (Basel). 2020 Jun 28;10(7):1107. doi: 10.3390/ani10071107 (PMC7401521; doi:10.3390/ani10071107)
Supplement: Supplementary file 1 [file animals-10-01107-s001.pdf]

Supplementary Information

Title of Manuscript:

Emotion Recognition in Cats

Authors:

Angelo Quaranta *, Serenella d'Ingeo, Rosaria Amoruso and Marcello Siniscalchi

Department of Veterinary Medicine, Animal Physiology and Behavior Unit, University of Bari "Aldo Moro", 70121 Bari, Italy; serenella.dingeo@uniba.it (S.d.); rosy.amoruso@gmail.com (R.A.); marcello.siniscalchi@uniba.it (M.S.)

*Correspondence: angelo.quaranta@uniba.it, Tel.: +39-080-544-3927

**Behavioral Category**	**Scored Behavior**
Stress/Anxiety	ears held in tension<br>slightly spatulate tongue<br>tongue way out<br>braced legs<br>tail down-tucked<br>panting<br>salivating<br>look away of avoidance<br>flattended ears<br>head lowered<br>paw lifted<br>lowering of the body posture<br>vocalization<br>shaking of the body<br>running away<br>hiding<br>freezing<br>seeking attention from the owner<br>head turn<br>turn away

Supplementary Table 1. List of behaviors scored according to the Stress/Anxiety category [1].

References

1. Bradshaw, J.W. The Behaviour of the Domestic Cat; Cabi: Wallingford, Oxfordshire, UK, 2012. ISBN 1845939921.
